# White Matter Tract Integrity and Cognitive, Emotional, and Social Outcomes After Acquired Brain Injury: Exploratory Tractography Findings for Personalized Neurorehabilitation

**DOI:** 10.3390/life15121849

**Published:** 2025-12-01

**Authors:** Rosario Bordón-Guerra, Eilin Ferreiro-Díaz-Velis, Coralia Sosa-Pérez, Sara Bisshopp-Alfonso, José Luis Hernández-Fleta, Jesús Morera-Molina, Wenceslao Peñate-Castro

**Affiliations:** 1 Department of Psychiatry, Hospital Universitario de Gran Canaria Doctor Negrín, 35010 Las Palmas de Gran Canaria, Spain; eferdiaj@gobiernodecanarias.org; 2 Faculty of Health Sciences, University of Las Palmas de Gran Canaria (ULPGC), 35001 Las Palmas de Gran Canaria, Spain; 3 Department of Neurosurgery, Hospital Universitario de Gran Canaria Doctor Negrín, 35010 Las Palmas de Gran Canaria, Spain; coralia.sosa@gmail.com (C.S.-P.); sarabisshop@gmail.com (S.B.-A.); jmormol@gobiernodecanarias.org (J.M.-M.); 4 Department of Medical and Surgical Sciences, Psychiatry and Medical Psychology Knowledge Area, University of Las Palmas de Gran Canaria, 35001 Las Palmas de Gran Canaria, Spain; jherfle@gobiernodecanarias.org; 5 Department of Clinical Psychology, Psychobiology and Methodology, Faculty of Psychology, University of La Laguna, 38200 La Laguna, Spain; wpenate@ull.es

**Keywords:** acquired brain injury, tractography, white matter tracts, cognition, empathy, neurorehabilitation

## Abstract

**Background:** Acquired brain injury (ABI) leads to cognitive, emotional, and social impairments that substantially affect quality of life. Although cortical lesions have traditionally received more attention, increasing evidence highlights the importance of the integrity of major white matter association tracts. However, few studies have simultaneously examined cognitive, affective, and social domains within a tractography framework. **Methods:** In this exploratory pilot study, ten ABI patients underwent diffusion-based tractography of the principal association tracts—the superior and inferior longitudinal fasciculi, the uncinate fasciculus, the inferior fronto-occipital fasciculus, and the cingulum—together with a comprehensive neuropsychological battery covering global cognition, executive functions, memory, emotional symptoms, and empathy. **Results:** Marked interindividual variability was observed in both tract profiles and neuropsychological outcomes. Findings revealed paradoxical associations, such as larger volumes of the left superior longitudinal fasciculus being linked to poorer cognitive performance, suggesting maladaptive reorganization. Hemispheric lateralization patterns were also identified, with the uncinate fasciculus showing differential contributions to immediate memory and working memory across hemispheres. Notably, empathy scores consistently correlated with volumes of the inferior longitudinal fasciculus, the uncinate fasciculus, and the cingulum, in line with recent evidence on the structural basis of socio-emotional outcomes after ABI. **Conclusions:** Although limited by sample size, this study provides novel evidence regarding the structure–function paradox, hemispheric specialization, and the clinical relevance of empathy in ABI. Overall, the results support the integration of tractography of the main association tracts with neuropsychological assessment as complementary tools to advance personalized neurorehabilitation.

## 1. Introduction

Acquired brain injury (ABI), including traumatic and vascular etiologies, often leads to persistent cognitive, emotional, and social sequelae that compromise autonomy and quality of life [1,2]. Beyond cortical damage, converging evidence indicates that white-matter disconnection is a major substrate of executive, attentional, and affective dysfunction [3,4,5]. Although patients may differ in lesion etiology, this study examines the shared structure–function relationships observed in mild ABI, focusing on how individual patterns of white-matter integrity relate to cognitive and emotional outcomes. Advances in diffusion MRI and tractography now make it possible to map association pathways in vivo, providing a window into patient-specific profiles of disconnection and recovery [6,7].

In parallel, artificial intelligence (AI) methods have begun to enhance neurorehabilitation research by integrating multimodal imaging and clinical data, thereby supporting personalized and predictive modeling [6,7,8]. Recent progress in precision connectomics and AI-driven tractography suggests that multimodal data fusion may improve prognostic accuracy and therapy selection in ABI. Within this framework, incorporating empathy and socio-emotional metrics could further refine predictive models of real-world recovery [9]. The present study represents an exploratory step toward AI-informed personalized neurorehabilitation in ABI.

Recent studies highlight the clinical relevance of tractography in ABI. Dynamic WM changes have been linked to late improvement or deterioration after TBI [9], tract-based analyses have predicted cognitive and emotional outcomes [10], and meta-analytic evidence underscores the role of structural disconnection in post-injury dysfunction [11]. Nevertheless, integrated investigations spanning cognition, emotion, and social cognition within the same tractography framework remain scarce, particularly regarding empathy and socio-emotional outcomes, which are highly relevant for neuropsychological rehabilitation.

Functionally, association pathways support distinct but complementary processes. The superior longitudinal fasciculus (SLF) comprises three major branches with fronto-parietal projections implicated in attention, working memory, and executive control [12]. The uncinate fasciculus (UF) links anterior temporal and orbitofrontal cortices and plays a role in episodic memory and affective regulation [13]. The inferior longitudinal fasciculus (ILF) supports visual–affective integration and socio-emotional processing [14], whereas the cingulum contributes to attentional control and affective regulation [15]. Recent evidence also suggests that WM microstructure in these tracts predicts outcomes in domains such as empathy, emotion regulation, and social cognition [16,17]. These findings help interpret the paradoxical associations often observed in ABI (e.g., larger tract volume associated with poorer performance), which may reflect maladaptive reorganization rather than preserved function [18].

Neuropsychological assessment provides complementary insights into tract–function associations. We selected a global cognition screener (MoCA [19]), processing speed and set-shifting (TMT-A/B [20]), working memory (Digit Span, WAIS-III [21]), visuoconstructive planning (Rey–Osterrieth Complex Figure [22]), affective symptoms (HADS [23]), and empathy as a key domain of social cognition (TECA [24]). This battery aligns with tract-based functional anatomy (e.g., SLF ↔ executive/visuospatial control; UF/cingulum ↔ emotional regulation; ILF/UF/cingulum ↔ socio-emotional processing) and targets rehabilitation-relevant outcomes.

Although diffusion-based tractography has previously been used to explore structure–function relationships after acquired brain injury [3,4,5], most studies have examined cognitive or affective domains in isolation, without considering their interaction with social processes. In particular, empathy, a key determinant of social reintegration and quality of life, has received little attention in tractography-based research [12,13,14,15,16]. The present exploratory pilot study addresses this gap by jointly analyzing cognitive, emotional, and social outcomes within a single tractography framework, offering a multidimensional perspective on structure–function associations that may inform personalized neurorehabilitation approaches.

Aims and hypotheses. This exploratory pilot study aimed to investigate associations between the volumes of the main association tracts (SLF, UF, ILF, IFOF, cingulum) and neuropsychological outcomes across cognition, emotion, and empathy. We expected tract-specific relationships consistent with their functional roles, including possible paradoxical associations reflecting maladaptive reorganization.

## 2. Materials and Methods

### 2.1. Participants

This cross-sectional pilot study was conducted between August 2022 and June 2023 at a tertiary hospital (Dr. Negrín of Gran Canaria, Spain). Patients were eligible if they: (1) had an ABI due to TBI or subarachnoid hemorrhage (SAH); (2) presented with cognitive or emotional sequelae mild enough to complete standardized testing but clinically significant to warrant evaluation; (3) were aged between 18 and 65 years, in order to minimize confounding effects of neurodevelopment or aging; and (4) had medical clearance for MRI. Exclusion criteria were: prior history of cognitive impairment, active substance use disorder, or contraindications for MRI.

Of the 15 patients initially screened, 5 were excluded (4 without neurocognitive impairment, 1 due to medical complications). The final sample consisted of 10 patients (6 women, 4 men; mean age = 51.1 years; range = 29–63). Given the feasibility constraints of diffusion-based tractography in a public hospital setting, small-N designs are common in this field. This study was therefore conceived as an exploratory, hypothesis-generating pilot aimed at identifying within-ABI structure–function associations rather than performing case–control or etiologic comparisons. This focus aligns with recent pilot studies in ABI using diffusion MRI with comparable sample sizes (8–15 participants) [25].

Consistent with contemporary models of acquired brain injury, which describe structural disconnection, network dysfunction, and reorganization processes as phenomena that often transcend the initial injury mechanism [3,4,5,6,16], patients with traumatic brain injury (TBI) and subarachnoid hemorrhage (SAH) were analyzed together without establishing etiopathogenic distinctions. In line with studies that prioritize the functional consequences of ABI over the specific etiology of the insult [1], the present work focuses on the neuropsychological and connectivity patterns derived from the acquired damage rather than on differences between etiological subgroups.

### 2.2. Neuropsychological Assessment

All participants underwent a standardized battery covering cognition, affect, and empathy:

- Montreal Cognitive Assessment (MoCA) [19]: a brief global cognition screener, sensitive to mild cognitive impairment, assessing multiple domains including attention, executive function, memory, language, and visuospatial abilities.
- Trail Making Test (TMT, Parts A and B) [20]: evaluates processing speed, visual attention, and cognitive flexibility. Part A requires sequencing numbers, while Part B assesses set-shifting between numbers and letters.
- Digit Span Forward and Backward (WAIS-III) [21]: measures attentional capacity, immediate memory, and working memory. The forward condition reflects attention span, while the backward condition indexes executive control and manipulation in working memory.
- Rey–Osterrieth Complex Figure Test (RCFT) [22]: only the copy task was administered, which evaluates visuoconstructive ability, perceptual organization, and planning strategies. Scoring considered both the global configuration and the details reproduced, yielding raw scores and percentile ranks.
- Hospital Anxiety and Depression Scale (HADS) [23]: a self-report instrument widely used for detecting anxiety and depressive symptoms in neurological populations.
- Cognitive and Affective Empathy Test (TECA) [24]: a validated Spanish instrument measuring empathy across four subscales, perspective taking, emotional understanding, empathic distress, and empathic joy, as well as a total empathy score.

Neuropsychological evaluations were conducted by a licensed clinical neuropsychologist to ensure methodological consistency.

### 2.3. MRI Acquisition and Tractography

MRI data were acquired using a clinical scanner with diffusion-weighted imaging sequences suitable for tractography. Standard pre-processing (motion and eddy-current correction) was applied, and major association tracts (SLF, ILF, UF, IFOF, cingulum) were reconstructed using deterministic fiber-tracking algorithms (Brainlab Elements, v2.0). Volumetric estimates (mm^3^) were obtained by summing voxels traversed by streamlines.

To obtain the tractography analysis, software developed by Brainlab was used (Elements Fibertracking, version 2.0). Deterministic streamline tracking was performed with the following stopping criteria: fractional anisotropy (FA) threshold = 0.20, maximum turning angle = 20°, voxel dimensions = 2.0 mm × 2.0 mm × 2.0 mm, and minimum fiber length = 80 mm. Tracking was terminated when any of these conditions were met or when streamlines exited the white-matter mask. Volumetric estimates (mm^3^) were obtained by summing voxels traversed by streamlines within each reconstructed tract. All acquisitions were obtained using the same scanner and protocol (b = 1000 s/mm^2^; 32 diffusion directions) to ensure methodological consistency across participants. All reconstructions were performed by an experienced neuroradiologist in collaboration with neurosurgeons, blinded to neuropsychological results. Fiber-tracking principles followed standard recommendations [26].

**Figure 1:**
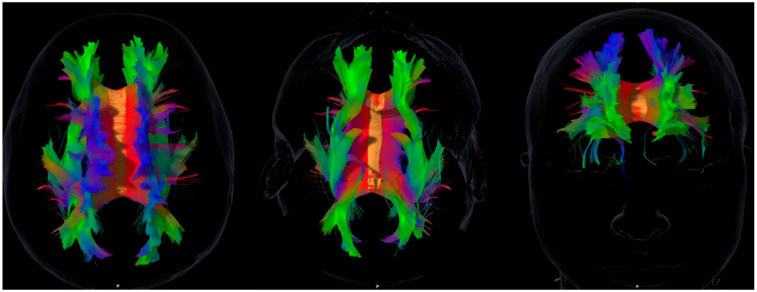
Representative diffusion tensor imaging (DTI) tractography showing the main association pathways analyzed in this study: superior and inferior longitudinal fasciculi (SLF, ILF), uncinate fasciculus (UF), inferior fronto-occipital fasciculus (IFOF), and cingulum. These reconstructions illustrate the anatomical substrates explored in relation to the cognitive, emotional, and social domains assessed. The figure is intended to provide a structural overview and does not imply direct or deterministic tract–function associations. Axial, sagittal, and coronal views are shown in native space for visual orientation.

The following association tracts were reconstructed: superior longitudinal fasciculus (SLF), inferior longitudinal fasciculus (ILF), uncinate fasciculus (UF), inferior fronto-occipital fasciculus (IFOF), and cingulum. The superior fronto-occipital fasciculus (SFOF) was also reconstructed. However, given the ongoing controversy about its existence in humans [27], SFOF volumes are reported for transparency but not emphasized in the main discussion or interpretation of results. Figure 1 provides an anatomical overview of the reconstructed association tracts included in the correlation analyses, illustrating the structural framework for the cognitive, emotional, and social domains explored.

### 2.4. Statistical Analyses

Descriptive statistics were used for demographic and clinical data. Normality of distributions was examined with the Shapiro–Wilk test. Given the small sample size and the exploratory nature of this pilot study, associations between tract volumes and neuropsychological outcomes were examined using Spearman’s rank correlation (two-tailed, *p* < 0.05). Primary analyses used uncorrected *p* < 0.05; for transparency, FDR-adjusted *p*-values and 95% CIs for correlations reaching uncorrected *p* < 0.05 are reported in Table 1. All analyses were conducted in R (Version 4.3.2; R Foundation for Statistical Computing, Vienna, Austria) and Python (Version 3.11; Scikit-learn v1.3.0, NetworkX v3.1, Matplotlib v3.8.0, Pandas v2.1.1).

Exploratory k-means clustering (k = 3) and simple network analysis were also conducted on the correlation matrix; nodes with |ρ| > 0.70 were considered hubs for descriptive purposes.

### 2.5. Ethics

Thisstudy was approved by the Institutional Ethics Committee (Hospital Dr. Negrín, Gran Canaria, Ref: CEIm 2022-206-1). Written informed consent was obtained from all participants, in line with the Declaration of Helsinki.

## 3. Results

Results are presented in five sections describing participant characteristics, neuropsychological outcomes, tract volumes, tract–behavior associations, and exploratory network analyses.

### 3.1. Participant Characteristics

Ten ABI patients (6 women, 4 men; mean age = 51.1 years, range = 29–63) were included. All presented with cognitive sequelae sufficient to warrant evaluation. Descriptive demographic and clinical data are presented in Table 2.

### 3.2. Neuropsychological Outcomes

All participants scored below the MoCA cutoff (<25), confirming global cognitive impairment [19]. Mean scores indicated consistent difficulties in attention and working memory (Digit Span forward = 5.4 ± 1.6; backward = 3.6 ± 0.7). Processing speed and flexibility (TMT A and B) were markedly heterogeneous, with several participants performing in the pathological range. RCFT scores reflected variable visuoconstructive and planning abilities.

Regarding emotional symptoms, mean HADS scores suggested mild-to-moderate anxiety (10.6 ± 4.3) and depression (8.9 ± 5.2). Social cognition, assessed via TECA, revealed reduced empathy across subdomains, particularly empathic distress and empathic joy.

### 3.3. White Matter Tract Volumes

Diffusion-based tractography successfully reconstructed all major association tracts (SLF, ILF, UF, IFOF, cingulum). Mean tract volumes are shown in Table 2.

### 3.4. Exploratory Tract–Behavior Associations

Overview. Spearman correlations revealed several significant associations between tract volumes and neuropsychological outcomes, summarized in Table 3.

The most notable findings involved the left SLF, which correlated negatively with MoCA (ρ = −0.64, *p* = 0.046) and RCFT (ρ = −0.66, *p* = 0.039) and positively with slower TMT performance (ρ = 0.86, *p* = 0.001). The left UF showed a negative correlation with Digit Span forward (ρ = −0.66, *p* = 0.038), whereas the right UF correlated positively with Digit Span backward (ρ = 0.68, *p* = 0.029). The left ILF, left cingulum, and right IFOF correlated negatively with empathy subscales, particularly empathic distress and empathic joy.

Additional findings. No significant associations were observed for HADS scores. Age correlated negatively with left cingulum volume (ρ = −0.73, *p* = 0.016). All results should be interpreted as exploratory, and FDR-adjusted *p*-values with 95% CIs are provided in Table 1.

### 3.5. Cluster and Network Analyses

Cluster analysis (k = 3) identified subgroups with distinct profiles. Network analysis highlighted the left SLF and bilateral UF as structural hubs (|ρ| > 0.7):

- Cluster 1: marked cognitive impairment with preserved empathy.
- Cluster 2: mixed cognitive and affective deficits.
- Cluster 3: milder deficits but reduced empathy across domains.

## 4. Discussion

This exploratory pilot study examined associations between white matter tract integrity and cognitive, emotional, and social outcomes in patients with acquired brain injury (ABI). The results revealed paradoxical structure–function associations, consistent with compensatory or maladaptive mechanisms of reorganization, and highlighted the role of specific tracts in supporting empathy and other socio-emotional functions.

### 4.1. Structure–Function Paradox and Network Reorganization

One of the most striking findings was the negative association between tract volumes and performance in cognitive tasks, particularly for the left superior longitudinal fasciculus (SLF). Larger SLF volumes correlated with poorer global cognition (MoCA), reduced visuospatial organization (RCFT), and slower executive performance (TMT). These paradoxical associations challenge the assumption that larger structural volumes necessarily imply better function. Instead, they may reflect inefficient or maladaptive reorganization, where volumetric increases occur without corresponding microstructural efficiency [18].

Similar findings have been reported in post-stroke and aging populations, where volumetric hypertrophy coexists with functional decline [3,28]. Such evidence reinforces the perspective that recovery is determined less by absolute tract size and more by network integration and microstructural quality [4,5]. These findings expand upon classical models of brain plasticity that describe the coexistence of adaptive and maladaptive reorganization after injury [29]. In line with recent mechanism-aware perspectives, they can also be interpreted within the contemporary damage-versus-defence framework [30], suggesting that volumetric enlargement after injury may represent self-defensive or compensatory remodeling—such as glial proliferation or inefficient collateralization—aimed at maintaining function despite reduced microstructural quality. From this perspective, paradoxical associations between tract volume and behavioral performance may reflect the brain’s attempt to preserve connectivity within reorganized, yet less efficient, networks.

### 4.2. Lateralization and Tract-Specific Contributions

Thisstudy also revealed hemispheric asymmetries. The left UF correlated negatively with immediate memory (Digit Span forward), whereas the right UF correlated positively with working memory (Digit Span backward). These findings align with lateralization models, suggesting that left tracts support verbal–executive functions while right tracts contribute more strongly to socio-emotional and memory regulation.

These results are consistent with tractography studies linking the UF and cingulum to memory and emotion, and the SLF to executive control [12,13]. Such asymmetries highlight the need for individualized rehabilitation, as patients may present with lateralized vulnerabilities requiring targeted interventions.

### 4.3. Social Cognition and Empathy as Novel Contributions

A distinctive contribution of this study is the inclusion of empathy. Few tractography studies in ABI have directly examined social cognition, despite its critical role in quality of life and reintegration. Our results revealed consistent associations between empathy (TECA scores) and ILF, UF, and cingulum volumes. Finally, our findings align with broader evidence linking social and emotional engagement with cognitive and functional resilience across the lifespan. This reserve-based perspective reinforces the clinical relevance of empathy as a potential target for neurorehabilitation and as a marker of adaptive recovery capacity after ABI [31].

The negative correlations observed suggest that volumetric remodeling may represent compensatory mechanisms that sustain socio-emotional performance through alternative pathways. This aligns with prior evidence implicating fronto-limbic and temporo-occipital networks in empathy and socio-emotional processing [16,17]. By demonstrating that empathy can be structurally anchored in ABI, this study expands the scope of neurorehabilitation beyond executive and motor domains.

### 4.4. Clinical Implications

Clinically, these findings underscore the potential of tractography to complement neuropsychological assessment. Two patients with comparable test scores may differ substantially in tract integrity, which could explain divergent rehabilitation trajectories. Identifying such tract-specific profiles may help clinicians tailor interventions: for example, prioritizing attentional and executive training in patients with SLF involvement, or incorporating emotional regulation therapies when UF or cingulum asymmetries are evident.

This perspective supports the emerging paradigm of precision neurorehabilitation, in which interventions are individualized based on residual structural and functional connectivity rather than group averages [4]. Novel experimental approaches in TBI rehabilitation further underscore the need for individualized, multimodal strategies that extend beyond cognitive training alone [32].

### 4.5. Future Directions

While exploratory, this study lays the foundation for future multimodal investigations. Larger longitudinal cohorts should confirm whether the associations reported here persist over time and predict recovery. Integrating tractography with advanced microstructural metrics (fixel-based analysis, NODDI), functional modalities (resting-state fMRI, EEG), and connectome-based modeling will be critical to capture dynamic reorganization [15].

Artificial intelligence methods are increasingly being applied to multimodal neuroimaging and may enable predictive models that combine structural, functional, and clinical variables to forecast rehabilitation outcomes [6,7,8]. Incorporating empathy and social cognition into such models could help optimize not only functional but also social reintegration.

### 4.6. Limitations

Several limitations must be acknowledged. First, the small sample size constrains statistical power and generalizability. Nevertheless, sample sizes in tractography studies of ABI often range between 8 and 15 patients due to the logistical complexity and costs of acquisition [25,28]. Accordingly, this work should be interpreted as hypothesis-generating rather than confirmatory.

Second, the absence of a control group limits between-group comparisons; however, this study was intentionally designed to characterize intra-individual variability, which is highly relevant for personalized neurorehabilitation. Additionally, deterministic tracking parameters (FA = 0.20; maximum turning angle = 20°; voxel size ≈ 2 mm; minimum fiber length = 80 mm) may have limited the reconstruction of fibers with high curvature or low anisotropy, such as limbic pathways (e.g., cingulum or uncinate fasciculus). This limitation is inherent to clinical deterministic tractography and was considered when interpreting the results.

Third, because multiple correlations were explored, there is an increased risk of Type I error; to mitigate this, FDR-adjusted values are provided in Table 1 and results are interpreted as exploratory, future larger-scale studies will be required to confirm these preliminary associations.

The inclusion of mixed etiologies (TBI and SAH) also represents a relevant limitation that may reduce the generalizability of the findings. Despite heterogeneity is common in studies focused on the functional impact and network reorganization following acquired brain injury, regardless of its initial cause [1,3,4], the results presented here should be interpreted with caution. Future research with larger and etiologically homogeneous cohorts will be required to confirm these preliminary observations.

Volumetric indices provide indirect estimates of tract integrity; combining tractography with advanced microstructural (e.g., NODDI, fixel-based analysis) and functional methods will be essential in subsequent work.

Finally, although SFOF volumes were reconstructed and reported in the results tables, their interpretation should be considered tentative. The existence of the SFOF in humans remains debated, with some studies supporting its identification and others suggesting that it may reflect spurious reconstruction in tractography [27]. Given this controversy and the small sample size, SFOF data are provided for transparency but were not emphasized in the discussion.

## 5. Conclusions

This exploratory pilot study suggests that associations between white matter tract volumes and cognitive, affective, and empathy-related outcomes in acquired brain injury may be more complex than traditionally assumed. Preliminary findings point to the involvement of association tracts such as the ILF, UF, and cingulum in empathy and emotional regulation, extending the discussion of rehabilitation targets beyond conventional cognitive domains. These results should be interpreted with caution, yet they support the feasibility of integrating tractography with neuropsychological assessment and provide hypotheses for future research with larger samples to validate and expand these observations.

## Figures and Tables

**Table 1:** FDR-adjusted correlation results between major white matter tracts and neuropsychological measures in patients with acquired brain injury.

Tract–Test	ρ	Uncorrected *p*	FDR-Adjusted *p*	95% CI for ρ	Interpretation
Left SLF—MoCA	−0.64	0.046	0.052	[−0.91, −0.04]	Trend
Left SLF—RCFT	−0.66	0.039	0.059	[−0.92, −0.06]	Trend
Left SLF—TMT (B)	0.86	0.001	0.009	[0.48, 0.97]	**Significant**
Left UF—Digit Span Fw	−0.66	0.038	0.068	[−0.91, −0.07]	Trend
Right UF—Digit Span Bw	0.68	0.029	0.065	[0.09, 0.93]	Trend
Left ILF—Empathy Total	−0.66	0.039	0.059	[−0.91, −0.07]	Trend
Left Cingulum—Empathic Distress	−0.64	0.046	0.052	[−0.91, −0.04]	Trend
Right IFOF—Empathic Joy	−0.71	0.022	0.066	[−0.94, −0.12]	Trend
Age—Left Cingulum	−0.73	0.016	0.072	[−0.94, −0.17]	Trend

FDR adjusted using Benjamini–Hochberg; CI computed for n = 10, α = 0.05. Results are exploratory and should be interpreted with caution.

**Table 2:** Patient characteristics, test scores, and volumes of association tracts.

		N = 10
Mean age (range)		51.1 (29–63)
Proportion of women		60%
Social Cognition, Cognitive Functions, And Emotional Outcomes		
Montreal Cognitive Assessment test. Mean score (SD)		21.9 (3.03)
Digit Span Test. Mean score (SD)	Forward	5.4 (1.6)
	Reverse	3.6 (0.7)
Trail Making Test. Mean time, seconds (SD)	Part A	51.3 (28.6)
	Part B	147.5 (173.3)
Rey Complex Figure Test. Mean score (SD)	Type	42.0 (26.5)
	Percentile (PC)	60.0 (25.8)
Hospital Anxiety and Depression Scale. Mean score (SD)	Depression	8.9 (5.2)
	Anxiety	10.6 (4.3)
Empathy Test. Mean score (SD)	Total	38.8 (32.4)
	Perspective taking	33.5 (27.7)
	Emotional understanding	30.1 (24.4)
	Empathic distress	43.1 (31.4)
	Empathic joy	33.7 (25.6)
Volumes of Association Tracts		Mean volume mm^3^ (SD)
Fronto-occipital	Right superior	501.8 (100.9)
	Left superior	518.8 (89.7)
	Right inferior	525.5 (60.8)
	Left inferior	524.5 (66.2)
Uncinate fasciculus	Right	392.6 (130.6)
	Left	400.8 (142.3)
Longitudinal fasciculus	Right superior	425.2 (111.9)
	Left superior	418.2 (85.7)
	Right inferior	515.2 (78.4)
	Left inferior	496.4 (76.9)
Cingulum	Right	461.7 (71.8)
	Left	495.8 (88.7)

Abbreviations: SD, standard deviation.

**Table 3:** Spearman’s correlation coefficient between test scores and volumes of association tracts.

Tract/Test	MoCA	DST		TMT		RCFT		HADS		TECA				
		Fw	Rv	Part A	Part B	TIPO	PC	Dep	Anx	Total	PT	EU	ED	EJ
Right superior fronto-occipital	−0.13 (0.724)	0.06 (0.862)	0.09 (0.811)	−0.18 (0.629)	−0.16 (0.663)	−0.09 (0.797)	0.11 (0.772)	−0.37 (0.287)	−0.1 (0.776)	−0.08 (0.827)	−0.23 (0.521)	−0.14 (0.699)	−0.61 (0.064)	0.00 (1.00)
Left superior fronto-occipital	−0.28 (0.431)	0.02 (0.958)	0.24 (0.502)	0.30 (0.399)	0.46 (0.179)	−0.38 (0.285)	−0.29 (0.413)	−0.12 (0.748)	−0.09 (0.815)	−0.40 (0.254)	−0.38 (0.275)	−0.07 (0.854)	−0.32 (0.370)	−0.26 (0.476)
Right inferior fronto-occipital	0.10 (0.787)	−0.53 (0.112)	0.19 (0.589)	0.27 (0.457)	0.05 (0.894)	0.09 (0.796)	0.32 (0.371)	0.09 (0.813)	0.03 (0.940)	−0.20 (0.586)	0.14 (0.693)	−0.37 (0.293)	−0.06 (0.866)	**−0.71 (0.022)**
Left inferior fronto-occipital	0.23 (0.528)	−0.39 (0.268)	−0.07 (0.846)	0.44 (0.208)	0.29 (0.422)	−0.19 (0.603)	0.13 (0.719)	−0.06 (0.866)	−0.26 (0.463)	−0.33 (0.358)	0.30 (0.397)	−0.30 (0.400)	−0.03 (0.940)	−0.54 (0.105)
Right uncinate fasciculus	0.43 (0.217)	−0.15 (0.688)	**0.68 (0.029)**	−0.35 (0.321)	−0.38 (0.275)	0.03 (0.932)	0.29 (0.413)	−0.03 (0.933)	0.05 (0.894)	0.01 (0.987)	0.29 (0.413)	−0.24 (0.508)	−0.24 (0.507)	**−0.69 (0.028)**
Left uncinate fasciculus	0.36 (0.306)	**−0.66 (0.038)**	0.60 (0.069)	−0.19 (0.592)	−0.25 (0.487)	0.34 (0.331)	0.55 (0.097)	0.30 (0.399)	0.16 (0.662)	−0.15 (0.672)	0.20 (0.578)	−0.48 (0.165)	−0.28 (0.441)	**−0.66 (0.037)**
Right superior longitudinal fasciculus	−0.31 (0.390)	−0.10 (0.781)	−0.18 (0.617)	0.58 (0.078)	0.18 (0.614)	−0.47 (0.172)	−0.28 (0.434)	−0.01 (0.987)	0.26 (0.464)	0.07 (0.853)	0.07 (0.841)	0.16 (0.662)	0.03 (0.933)	−0.24 (0.498)
Left superior longitudinal fasciculus	**−0.64 (0.046)**	−0.03 (0.944)	−0.44 (0.205)	**0.86 (0.001)**	**0.72 (0.020)**	−0.55 (0.099)	**−0.66 (0.039)**	0.15 (0.670)	0.19 (0.599)	0.06 (0.865)	−0.15 (0.679)	0.41 (0.238)	0.29 (0.412)	0.13 (0.711)
Right inferior longitudinal fasciculus	0.57 (0.082)	0.49 (0.147)	0.44 (0.201)	−0.49 (0.147)	−0.32 (0.374)	−0.34 (0.331)	−0.4 (0.247)	0.47 (0.168)	0.47 (0.171)	0.08 (0.827)	0.22 (0.544)	0.23 (0.531)	0.49 (0.151)	0.23 (0.521)
Left inferior longitudinal fasciculus	0.39 (0.263)	0.15 (0.688)	0.42 (0.224)	0.13 (0.731)	0.34 (0.336)	−0.22 (0.544)	−0.16 (0.668)	0.06 (0.880)	−0.22 (0.542)	**−0.66 (0.039)**	−0.06 (0.868)	−0.28 (0.432)	0.29 (0.410)	−0.58 (0.077)
Right cingulum	0.02 (0.946)	−0.48 (0.162)	**0.66 (0.039)**	−0.14 (0.703)	0.06 (0.873)	0.38 (0.282)	0.18 (0.616)	0.60 (0.067)	0.34 (0.331)	−0.06 (0.865)	−0.11 (0.756)	−0.16 (0.666)	0.03 (0.926)	−0.35 (0.328)
Left cingulum	−0.23 (0.523)	−0.28 (0.429)	0.46 (0.177)	0.03 (0.938)	−0.10 (0.776)	0.09 (0.796)	0.11 (0.771)	0.15 (0.671)	0.24 (0.513)	0.16 (0.659)	0.07 (0.847)	0.24 (0.507)	**−0.64 (0.046)**	−0.09 (0.815)

**Abbreviations:** Spearman’s correlation coefficients (ρ) between tract volumes and neuropsychological outcomes. Bold values indicate statistically significant correlations (*p* < 0.05). Abbreviations: Anx, anxiety; Dep, depression; DST, Digit Span test; ED, empathic distress; EJ, empathic joy; EU, emotional understanding; Fw, forward; HADS, Hospital Anxiety and Depression Scale; MoCA, Montreal Cognitive Assessment; PC, percentile score; PT, perspective taking; RCFT, Rey Complex Figure Test; Rv, reverse; TECA, Cognitive and Affective Empathy Test; TIPO, total drawing performance-adjusted percentile score; TMT, Trail Making Test. Bold values indicate uncorrected *p* < 0.05; corresponding FDR-adjusted *p*-values and 95% CIs are available in Table 1.

## Data Availability

The data presented in this study are available on reasonable request from the corresponding author. The data are not publicly available due to privacy restrictions.

## Abbreviations

The following abbreviations are used in this manuscript:

ABI	Acquired brain injury
Anx	Anxiety
Dep	Depression
DST	Digit Span Test
ED	Empathic distress
EJ	Empathic joy
EU	Emotional understanding
Fw	Forward (Digit Span)
HADS	Hospital Anxiety and Depression Scale
ILF	Inferior longitudinal fasciculus
IFOF	Inferior fronto-occipital fasciculus
MoCA	Montreal Cognitive Assessment
PC	Percentile score
PT	Perspective taking
RCFT	Rey–Osterrieth Complex Figure Test
Rv	Reverse (Digit Span)
SD	Standard deviation
SFOF	Superior fronto-occipital fasciculus
SLF	Superior longitudinal fasciculus
TECA	Cognitive and Affective Empathy Test
TIPO	Total drawing performance-adjusted percentile score
TMT	Trail Making Test
UF	Uncinate fasciculus
WAIS	Wechsler Adult Intelligence Scale
WM	White matter
ρ	Spearman’s correlation coefficient
